# Furstenberg Family and Chaos for Time-Varying Discrete Dynamical Systems

**DOI:** 10.3390/e26080674

**Published:** 2024-08-09

**Authors:** Risong Li, Yongjiang Li, Tianxiu Lu, Jiazheng Zhao, Jing Su

**Affiliations:** 1 School of Mathematic and Computer Science, Guangdong Ocean University, Zhanjiang 524025, China; lirs@gdou.edu.cn (R.L.); lyjriver@gdou.edu.cn (Y.L.); jingsu@gdou.edu.cn (J.S.); 2 College of Mathematics and Statistics, Sichuan University of Science and Engineering, Zigong 643000, China; 323070108114@stu.suse.edu.cn; 3 South Sichuan Applied Mathematics Research Center, Zigong 643000, China

**Keywords:** Furstenberg family, scrambled pair, scrambled set, generically G-scrambled time-varying discrete dynamical system

## Abstract

Assume that (Y,ρ) is a nontrivial complete metric space, and that $\left(Y,g_{1,\infty }\right)$ is a time-varying discrete dynamical system (T-VDDS), which is given by sequences ${\left(g_{l}\right)}_{l=1}^{\infty }$ of continuous selfmaps $g_{l}:Y\to Y$. In this paper, for a given Furstenberg family G and a given T-VDDS $\left(Y,g_{1,\infty }\right)$, G-scrambled pairs of points of the system $\left(Y,g_{1,\infty }\right)$ (which contains the well-known scrambled pairs) are provided. Some properties of the set of G-scrambled pairs of a given T-VDDS $\left(Y,g_{1,\infty }\right)$ are studied. Moreover, the generically G-chaotic T-VDDS and the generically strongly G-chaotic T-VDDS are defined. A sufficient condition for a given T-VDDS to be generically strongly G-chaotic is also presented.

## 1. Introduction

Li and Yorke [1] first presented the term “chaos”. Many scholars have since explored the Li–Yorke chaotic autonomous discrete systems (ADDSs), see, for example [1,2,3,4,5,6,7,8]. Schweizer and Smítal [9] defined another sort of chaos, which is called distributional chaos. Distributionally chaotic ADDSs have been explored greatly by lots of researchers at home and abroad (see [2,3,10,11]). In 2007, ref. [2] provided the definition of chaos via G, where G is a fixed Furstenberg family. Research has demonstrated that Li–Yorke chaos and various forms of distributional chaos can be characterized as chaos within Furstenberg families. Moreover, they explored some fundamental and important properties of the set of G-scrambled pairs of a given ADDS (Y,g) and provided the concepts of the generically G-chaotic ADDS and the generically strongly G-chaotic ADDS. They also determined a sufficient condition under which an ADDS can be considered generally highly chaotic. The concept of chaos, defined as $\left(G_{1},G_{2}\right)$, was introduced in 2009 by [3], using a pair of Furstenberg families denoted as $G_{1}$ and $G_{2}$. According to the findings of [3], both Li–Yorke chaos and distributional chaos fall under the umbrella of chaos within the context of Furstenberg families. In addition, they derived multiple sufficient conditions that determine if a system is $\left(G_{1},G_{2}\right)$-chaotic and constructed an interesting and useful example. Furthermore, it was revealed that while an uncountable scrambled set may be foreshadowing of the second type of distributional chaos, it does not necessarily imply the presence of the first type of distributional chaos.

T-VDDSs were introduced in [12]. We know that they also appear connected to some time-varying difference equations. Chaos of T-VDDSs has been extensively studied (see [12,13,14,15,16,17,18]). For a sequence ${\left\{g_{l}\right\}}_{l=1}^{\infty }$ of continuous selfmaps on [0,1] converging uniformly to a continuous selfmap *g* on [0,1]; in [14], J.S. Canovas studied the limit behaviour of the T-VDDS $g_{1,\infty }$ and whether the simplicity (resp. chaoticity) of *g* can deduce the simplicity (respectively chaoticity) of $g_{1,\infty }$. In [15], J. Dvorakova discussed a T-VDDS $g_{1,\infty }$ on [0,1], where ${\left\{g_{l}\right\}}_{l=1}^{\infty }$ converges uniformly to a continuous selfmap *g* on [0,1], and $g_{l}$ is a surjective continuous map of [0,1] for each integer l>0, and it is proven that for a given time-varying system $g_{1,\infty }$, the full Lebesgue measure of a distributional scrambled set of $g_{1,\infty }$ does not deduce the existence of distributional chaos of the limit selfmap *g* of ${\left\{g_{l}\right\}}_{l=1}^{\infty }$ and, conversely, that a time-varying system exists with an arbitrarily small distributional scrambled set converging to a map distributional chaotic a.e.

This work extends the results of Xiong et al. in [2] to T-VDDSs. In particular, for a given Furstenberg family and a given T-VDDS $\left(Y,g_{1,\infty }\right)$, we define G-scrambled pairs of points for the system $\left(Y,g_{1,\infty }\right)$, which contains well-known scrambled pairs. We also study some properties of the set of G-scrambled pairs of a given T-VDDS $\left(Y,g_{1,\infty }\right)$ and define the generically G-chaotic T-VDDSs and the generically strongly G-chaotic T-VDDSs. Moreover, we obtain a sufficient condition for a given T-VDDS to be generically strongly G-chaotic.

In Section 2, some notations and basic concepts are recalled. Moreover, for a T-VDDS $\left(Y,g_{1,\infty }\right)$ and a given Furstenberg family G, we define G-scrambled pairs of points for the system $\left(Y,g_{1,\infty }\right)$. In Section 3, our main results are established.

## 2. Preliminaries

Throughout this paper, *Y* is a complete metric space with a metric ρ, and $\left(Y,g_{1,\infty }\right)$ is assumed to be a T-VDDS given by sequences ${\left(g_{l}\right)}_{l=1}^{\infty }$ of continuous selfmaps $g_{l}:Y\to Y$ from the complete metric space (Y,ρ) to itself. Note that if $g_{l}=g$ for any integer l>0, then system (Y,g) is a “classical” ADDS.

For any C⊂Y with C≠∅, let $\bar{C}$ denote the closure of *C* and ${\left[C\right]}_{\tau }$ denote the set {y∈Y:ρ(y,C)<τ} for any τ>0. Let the diagonal $\Delta_{Y}=\left\{\left(y,y\right):y\in Y\right\}$ and $\tilde{\rho }$ be the product metric on the product space Y×Y, which is defined by

$$\tilde{\rho }\left(\left(u_{1},v_{1}\right),\left(u_{2},v_{2}\right)\right)=\max\left\{\rho \left(u_{1},u_{2}\right),\rho \left(v_{1},v_{2}\right)\right\}$$

for any $\left(u_{1},v_{1}\right),\left(u_{2},v_{2}\right)\in Y\times Y$.

For any pair (u,v)∈Y×Y and a given integer l>0, we provide a distribution function $\psi_{uv}^{\left(l\right)}:\left(-\infty ,+\infty \right)\to \left[0,1\right]$ for a given T-VDDS $\left(Y,g_{1,\infty }\right)$ by

$$\psi_{uv}^{\left(l\right)}\left(g_{1,\infty },t\right)=\frac{1}{l}\sum_{j=0}^{l-1}\chi_{\left[0,t\right)}\left(\rho \left(g_{1}^{j}\left(u\right),g_{1}^{j}\left(v\right)\right)\right).$$

Then, $\psi_{uv}^{\left(l\right)}\left(g_{1,\infty },t\right)$ is a non-decreasing function, $\psi_{uv}^{\left(l\right)}\left(g_{1,\infty },t\right)=0$ for t≤0 and $\psi_{uv}^{\left(l\right)}\left(g_{1,\infty },t\right)=1$ for any t>d(Y), where d(Y) is the diameter of *Y*. Let

$$\psi_{uv}\left(g_{1,\infty },t\right)=\underset{l\to \infty }{lim inf}\psi_{uv}^{\left(l\right)}\left(g_{1,\infty },t\right)$$

and

$$\psi_{uv}^{*}\left(g_{1,\infty },t\right)=\underset{l\to \infty }{lim sup}\phi_{uv}^{\left(l\right)}\left(g_{1,\infty },t\right).$$

Then, both functions $\psi_{uv}\left(g_{1,\infty },t\right)$ and $\psi_{uv}^{*}\left(g_{1,\infty },t\right)$ are nondecreasing. Clearly, $\psi_{uv}\left(g_{1,\infty },t\right)\leq \psi_{uv}^{*}\left(g_{1,\infty },t\right)$ for any t∈(−∞,+∞). If $\psi_{uv}\left(g_{1,\infty },t\right)<\psi_{uv}^{*}\left(g_{1,\infty },t\right)$ for all *t* in an interval, it is simply written as $\psi_{uv}\left(g_{1,\infty }\right)<\phi_{uv}^{*}\left(g_{1,\infty }\right)$.

A pair (u,v)∈Y×Y is called a scrambled pair in the distribution sense for a given T-VDDS $\left(Y,g_{1,\infty }\right)$ if $\psi_{uv}^{*}\left(g_{1,\infty }\right)\equiv 1$ and $\psi_{uv}\left(g_{1,\infty },\tau \right)=0$ for some τ>0.

For a given τ>0, a pair (u,v)∈Y×Y is called a τ-scrambled pair in the distribution sense for a given T-VDDS $\left(Y,g_{1,\infty }\right)$ if $\psi_{uv}^{*}\left(g_{1,\infty }\right)\equiv 1$ and $\psi_{uv}\left(g_{1,\infty },\tau \right)=0$.

A subset S⊂Y is called a scrambled set in the distribution sense for a given T-VDDS $\left(Y,g_{1,\infty }\right)$ if any two different points in *S* is a scrambled pair in the distribution sense for this system.

A subset S⊂Y is called a strongly scrambled set in the distribution sense for a given T-VDDS $\left(Y,g_{1,\infty }\right)$ if there is a τ>0, satisfying that any (u,v)∈S×S(u≠v) is a τ-scrambled pair in the distribution sense for this system.

A T-VDDS $\left(Y,g_{1,\infty }\right)$ is called Li–Yorke chaotic if there is an uncountable subset S⊂Y, such that for any pair (u,v)∈S×S with u≠v,

$$\underset{l\to \infty }{lim inf}\rho \left(g_{1}^{l}\left(u\right),g_{1}^{l}\left(v\right)\right)=0$$

and

$$\underset{l\to \infty }{lim sup}\rho \left(g_{1}^{l}\left(u\right),g_{1}^{l}\left(v\right)\right)>0.$$

For any given τ>0 (resp. τ=0), a pair (u,v)∈Y×Y is called a τ-scrambled pair (resp. scrambled pair) in the sense of Li and Yorke for a given T-VDDS $\left(Y,g_{1,\infty }\right)$ if

$$\underset{l\to \infty }{lim inf}\rho \left(g_{1}^{l}\left(u\right),g_{1}^{l}\left(v\right)\right)=0$$

and

$$\underset{l\to \infty }{lim sup}\rho \left(g_{1}^{l}\left(u\right),g_{1}^{l}\left(v\right)\right)>\tau .$$

A subset S⊂Y is called a scrambled set in the sense of Li and Yorke for a given T-VDDS $\left(Y,g_{1,\infty }\right)$ if any (u,v)∈S×S(u≠v) is a scrambled pair in the sense of Li and Yorke for this system.

A subset S⊂Y is called a strongly scrambled set in the sense of Li and Yorke for a given T-VDDS $\left(Y,g_{1,\infty }\right)$ if there is a τ>0 satisfying that any (u,v)∈S×S(u≠v) is a τ-scrambled pair in the sense of Li and Yorke for this system.

Let $Z^{+}=\left\{1,2,\cdots \right\}$ and $Q=\{G|G\in Z^{+}\}$. A collection G⊂Q is said to be a Furstenberg family if it is hereditary upwards, that is, $G_{1}\subset G_{2}$ and $G_{1}\in G$ implies $G_{2}\in G$.

Let B={B∈Q:|B|=+∞}, where |B| denotes the cardinality of a set *B*. Clearly, B is a Furstenberg family.

Let $I=\left\{k_{1}<k_{2}<\cdots \right\}\subset Z^{+}$ be an infinite subset, and let

$$\nu^{*}\left(I\right)=\underset{n\to \infty }{lim sup}\frac{\left|I\cap \{1,2,\cdots ,n\}\right|}{n},$$

then, $\nu^{*}\left(I\right)$ is the upper density of $I\subset Z^{+}$. It is clear that $\nu^{*}\left(I\right)=\underset{j\to \infty }{lim sup}\frac{j}{k_{j}}$.

For any a∈[0,1], let

$$\bar{M}\left(a\right)=\left\{G\in B:\nu^{*}\left(G\right)\geq a\right\}.$$

Clearly, $\bar{M}\left(0\right)=B$.

For a given T-VDDS $\left(Y,g_{1,\infty }\right)$, any B⊂Y and any y∈Y, we write

$$N\left(y,B,g_{1,\infty }\right)=\left\{k\in Z^{+}:g_{1}^{k}\left(y\right)\in B\right\}.$$

For a given Furstenberg family G and B⊂Y with B≠∅, a point y∈Y is an G-attaching point of *B* for a given T-VDDS $\left(Y,g_{1,\infty }\right)$ if $N(y,B,g_{1,\infty })\in G$, and that

$$G\left(B,g_{1,\infty }\right)=\underset{G\in G}{⋃}\underset{k\in G}{⋂}f_{1}^{-k}\left(B\right)$$

is the G-attaching set of *B* for the system $\left(Y,g_{1,\infty }\right)$. Obviously, $G(B,g_{1,\infty })$ is the set of all G-attaching points of *B* for the system $\left(Y,g_{1,\infty }\right)$. A point y∈Y is an G-adherent point of *B* for a given T-VDDS $\left(Y,g_{1,\infty }\right)$, if $N(y,{\left[B\right]}_{\tau },G_{1,\infty })\in G$ for any τ>0. Let

$$\alpha_{G}\left(B,g_{1,\infty }\right)=\left\{y\in Y:N\left(y,{\left[B\right]}_{\tau },g_{1,\infty }\right)\in G,\forall \tau >0\right\}.$$

A point y∈Y is called an G-adherent point of a given set B⊂Y for a given T-VDDS $\left(Y,g_{1,\infty }\right)$ if *y* is an G-attaching point of the set ${\left[B\right]}_{\tau }$ for this system. For a given τ>0, a point y∈Y is called an G-τ-escape point of a given set B⊂Y for a given T-VDDS $\left(Y,g_{1,\infty }\right)$, *y* is an G-attaching point of the set $y-\bar{{\left[B\right]}_{\tau }}$ for this system. A point y∈Y is called an G-escape point of a given set B⊂Y for a given T-VDDS $\left(Y,g_{1,\infty }\right)$ if *y* is an G-attaching point of the set $Y-\bar{{\left[B\right]}_{\tau }}$ for some τ>0 and this system. A point y∈Y is called an G-reciprocating point of a given set B⊂Y for a given T-VDDS $\left(Y,g_{1,\infty }\right)$ if it is both an G-adherent point and an G-escape point of the set *B* for this system. A point y∈Y is said to be an G-τ-reciprocating point of a given set B⊂Y for a given T-VDDS $\left(Y,g_{1,\infty }\right)$ if it is both an G-adherent point and an G-τ-escape point of the set *B* for this system. Let $\alpha_{G}\left(B,g_{1,\infty }\right)$ (resp. $\epsilon_{G}\left(B,g_{1,\infty }\right)$, $\epsilon_{G}\left(B,\delta ,g_{1,\infty }\right)$, $\theta_{G}\left(B,g_{1,\infty }\right)$ or $\theta_{G}\left(B,\delta ,g_{1,\infty }\right)$) be the set of G-adherent points (resp. G-escape points, G-τ-escape points, G-reciprocating points or G-τ-reciprocating points) of a given set B⊂Y for a given T-VDDS $\left(Y,g_{1,\infty }\right)$. Clearly, from the definitions, one has

$$\alpha_{G}\left(B,g_{1,\infty }\right)=\underset{\tau >0}{⋂}G\left({\left[B\right]}_{\tau },g_{1,\infty }\right)=\overset{\infty }{\underset{l=1}{⋂}}G\left({\left[B\right]}_{\frac{1}{l}},g_{1,\infty }\right),$$

$$\epsilon_{G}\left(B,\tau ,g_{1,\infty }\right)=G\left(Y-\bar{{\left[B\right]}_{\tau }},g_{1,\infty }\right),$$

$$\epsilon_{G}\left(B,g_{1,\infty }\right)=\underset{\tau >0}{⋃}\epsilon_{G}\left(B,\tau ,g_{1,\infty }\right),$$

$$\theta_{G}\left(B,\tau ,g_{1,\infty }\right)=\alpha_{G}\left(B,g_{1,\infty }\right)\cap \epsilon_{G}\left(B,\tau ,g_{1,\infty }\right)$$

and

$$\theta_{G}\left(B,g_{1,\infty }\right)=\alpha_{G}\left(B,g_{1,\infty }\right)\cap \epsilon_{G}\left(B,g_{1,\infty }\right).$$

For a given T-VDDS $\left(Y,g_{1,\infty }\right)$, an G-reciprocating point (resp. G-τ-reciprocating point) of the diagonal $\Delta_{Y}$ for the product system $\left(Y\times Y,g_{1,\infty }\times g_{1,\infty }\right)$ is called an G-scrambled pair (resp. G-τ-scrambled pair) of the system $\left(Y,g_{1,\infty }\right)$. A subset S⊂Y is called an G-scrambled scrambled set for a given T-VDDS $\left(Y,g_{1,\infty }\right)$ if any (u,v)∈S×S(u≠v) is an G-scrambled pair of this system. A subset S⊂Y is called a strongly G-scrambled scrambled set for a given T-VDDS $\left(Y,g_{1,\infty }\right)$ if there is a τ>0 such that any two different points in *S* is an G-τ-scrambled pair of this system. A given T-VDDS $\left(Y,g_{1,\infty }\right)$ is called generically G-chaotic if the set of all G-scrambled pairs of this system is a $G_{\delta }$ set, which is dense in Y×Y. A given T-VDDS $\left(Y,g_{1,\infty }\right)$ is called generically strongly G-chaotic if there is a τ>0, satisfying that the set of all G-τ-scrambled pairs of this system is a $G_{\delta }$ set, which is dense in Y×Y.

## 3. Main Results

In this section, some results of Xiong et al. in [2] are extended to T-VDDSs.

### 3.1. Furstenberg Families Compatible with a T-VDDS

For a given T-VDDS $\left(Y,g_{1,\infty }\right)$ and any A,B⊂Y, we write

$$N_{g_{1,\infty }}\left(A,B\right)=\left\{k\in Z^{+}:B\cap {\left(g_{1}^{k}\left(A\right)\right)}^{-1}\neq \emptyset \right\}=\left\{k\in Z^{+}:g_{1}^{k}\left(B\right)\cap A\neq \emptyset \right\}.$$

If A={y}, we write $N_{g_{1,\infty }}\left(y,B\right)=N_{g_{1,\infty }}\left(A,B\right)$. For a given Furstenberg family G and B⊂Y with B≠∅, a point y∈Y is an G-attaching point of *B* for a given T-VDDS $\left(Y,g_{1,\infty }\right)$ if $N_{g_{1,\infty }}\left(y,B\right)\in G$, and

$$G\left(B,g_{1,\infty }\right)=\underset{G\in G}{⋃}\underset{k\in G}{⋂}{\left(g_{1}^{k}\left(B\right)\right)}^{-1}$$

is the G-attaching set of *B* for the system $\left(Y,g_{1,\infty }\right)$.

**Theorem** **1.** *Assume that G is a Furstenberg family, and that $\left(Y,g_{1,\infty }\right)$ is a T-VDDS and B⊂Y. Then, we have*

$$G\left(B,g_{1,\infty }\right)=\underset{G\in \kappa G}{⋂}\underset{k\in G}{⋃}{\left(g_{1}^{k}\left(B\right)\right)}^{-1}.$$

**Proof.**  Clearly,

$$y\in \underset{G\in \kappa G}{⋂}\underset{k\in G}{⋃}{\left(g_{1}^{k}\left(B\right)\right)}^{-1}$$,

if and only if for any G∈κG, one can find k∈F with $y\in {\left(g_{1}^{k}\left(B\right)\right)}^{-1}$, if and only if $N_{g_{1,\infty }}\left(y,B\right)\in \kappa \kappa G=G$. So,

$$\underset{G\in \kappa G}{⋂}\underset{k\in G}{⋃}{\left(g_{1}^{k}\left(B\right)\right)}^{-1}\subset G\left(B,g_{1,\infty }\right).$$

Assume that

$$y\in G(B,g_{1,\infty }).$$

Then,

$$y\in \underset{G\in G}{⋃}\underset{k\in G}{⋂}{\left(g_{1}^{k}\left(B\right)\right)}^{-1}$$,

if and only if there is a G∈κG with $y\in {\left(g_{1}^{k}\left(B\right)\right)}^{-1}$ for any k∈G, if and only if there is a G∈κG such that $N_{g_{1,\infty }}\left(y,B\right)⊃G$. So,

$$\underset{G\in \kappa G}{⋂}\underset{k\in G}{⋃}{\left(g_{1}^{k}\left(B\right)\right)}^{-1}⊃G\left(B,g_{1,\infty }\right).$$

Thus, the proof is finished. □

A Furstenberg family G is called compatible with a given T-VDDS $\left(Y,g_{1,\infty }\right)$, if for any given open set F⊂Y, the set $G(F,g_{1,\infty })$ is a $G_{\delta }$ set.

**Theorem** **2.** *Assume that G is a Furstenberg family, and that $\left(Y,g_{1,\infty }\right)$ is a T-VDDS. If κG is countably generated and proper or if $G=\bar{M}\left(t\right)$ for some t∈[0,1], then the family G is compatible with the system $\left(Y,g_{1,\infty }\right)$.*

**Proof.**  Assume that F⊂Y is open. If κG is countably generated and proper, from the definition, there is C⊂Q, which generates κG. From Theorem 1,

$$G\left(F,g_{1,\infty }\right)=\underset{G\in \kappa G}{⋂}\underset{k\in G}{⋃}{\left(g_{1}^{k}\left(F\right)\right)}^{-1}=\underset{G\in C}{⋂}\underset{k\in G}{⋃}{\left(g_{1}^{k}\left(F\right)\right)}^{-1}.$$

This means $G(F,g_{1,\infty })$ is a $G_{\delta }$ set.

Assume that $G=\bar{M}\left(t\right)$ for some t∈[0,1]. Clearly, the theorem holds for t=0. For any t∈(0,1] and any open set F⊂Y, we have

$$\begin{array}{cc} & G\left(F,g_{1,\infty }\right)=\left\{y\in Y:\underset{m\to \infty }{lim sup}\frac{\left|\left\{j\in Z^{+}:y\in {\left(g_{1}^{j}\left(F\right)\right)}^{-1},1\leq j\leq m,m\in Z^{+}\right\}\right|}{m}\geq t\right\}\\ = & \left\{y\in Y:\underset{m\to \infty }{lim sup}\frac{\left|\left\{j\in Z^{+}:y\in {\left(g_{1}^{j}\left(F\right)\right)}^{-1},1\leq j\leq m,m\in Z^{+}\right\}\right|}{m}>t-\frac{1}{k},k\in Z^{+}\right\}. \end{array}$$

Obviously, for any $k,n\in Z^{+}$ and any t∈(0,1] there are positive integers m>n and l∈{1,2,⋯,m} such that $t-\frac{1}{k}<\frac{l}{m}\leq t$. So,

$$\left\{y\in Y:\underset{p\to \infty }{lim sup}\frac{\left|\left\{j\in Z^{+}:y\in {\left(g_{1}^{j}\left(F\right)\right)}^{-1},1\leq j\leq p,p\in Z^{+}\right\}\right|}{m}\geq \frac{l}{m},k\in Z^{+}\right\}$$

implies that there are positive integers $r_{1},r_{2},\cdots ,r_{l}$ with $1\leq r_{1}<r_{2}<\cdots <r_{l}\leq m$ and $g_{1}^{r_{i}}\left(y\right)\in F$ for any i∈{1,2,⋯,l}. Consequently,

$$G\left(F,g_{1,\infty }\right)=\underset{k\in Z^{+}}{⋂}\underset{n\in Z^{+}}{⋂}\overset{\infty }{\underset{m=n+1}{⋃}}\underset{l\in \Theta_{m}}{⋃}\underset{\left(r_{1},r_{2},\cdots ,r_{l}\right)\in \Delta_{l,m}}{⋃}\overset{l}{\underset{i=1}{⋂}}{\left(g_{1}^{r_{i}}\left(F\right)\right)}^{-1},$$

where

$$\Theta_{m}=\left\{l\in Z_{+}:t-\frac{1}{k}<\frac{l}{m}\leq t,1\leq l\leq m,m\in Z_{+}\right\}$$

and

$$\Delta_{l,m}=\left\{\left(r_{1},r_{2},\cdots ,r_{l}\right):1\leq r_{1}<r_{2}<\cdots <r_{l}\leq m,m\in Z_{+}\right\}.$$

This means that $G(F,g_{1,\infty })$ is a $G_{\delta }$ set. Thus, the proof is finished. □

### 3.2. The Sets of G-Reciprocating Points for a T-VDDS

A point y∈Y is called an G-adherent point of a given set B⊂Y for a given T-VDDS $\left(Y,g_{1,\infty }\right)$, if for any τ>0, *y* is an G-attaching point of the set ${\left[B\right]}_{\tau }$ for this system. For a given τ>0, a point y∈Y is called an G-τ-escape point of a given set B⊂Y for a given T-VDDS $\left(Y,g_{1,\infty }\right)$, *y* is an G-attaching point of the set $Y-\bar{{\left[B\right]}_{\tau }}$ for this system. A point y∈Y is called an G-escape point of a given set B⊂Y for a given T-VDDS $\left(Y,g_{1,\infty }\right)$ if *y* is an G-attaching point of the set $Y-\bar{{\left[B\right]}_{\tau }}$ for some τ>0 and this system. A point y∈Y is called an G-reciprocating point of a given set B⊂Y for a given T-VDDS $\left(Y,g_{1,\infty }\right)$ if it is both an G-adherent point and an G-escape point of the set *B* for this system. A point y∈Y is called an G-τ-reciprocating point of a given set B⊂Y for a given T-VDDS $\left(Y,g_{1,\infty }\right)$ if it is both an G-adherent point and an G-τ-escape point of the set *B* for this system. Let $\alpha_{G}\left(B,g_{1,\infty }\right)$ (resp. $\epsilon_{G}\left(B,g_{1,\infty }\right)$, $\epsilon_{G}\left(B,\tau ,g_{1,\infty }\right)$, $\theta_{G}\left(B,g_{1,\infty }\right)$ or $\theta_{G}\left(B,\tau ,g_{1,\infty }\right)$) be the set of G-adherent points (resp. G-escape points, G-τ-escape points, G-reciprocating points or G-τ-reciprocating points) of a given set B⊂Y for a given T-VDDS $\left(Y,g_{1,\infty }\right)$. Clearly, from the definitions, we have

$$\alpha_{G}\left(B,g_{1,\infty }\right)=\underset{\tau >0}{⋂}G\left({\left[B\right]}_{\tau },g_{1,\infty }\right)=\overset{\infty }{\underset{k=1}{⋂}}G\left({\left[B\right]}_{\frac{1}{k}},g_{1,\infty }\right),$$

$$\epsilon_{G}\left(B,\tau ,g_{1,\infty }\right)=G\left(Y-\bar{{\left[B\right]}_{\tau }},g_{1,\infty }\right),$$

$$\epsilon_{G}\left(B,g_{1,\infty }\right)=\underset{\tau >0}{⋃}\epsilon_{G}\left(B,\tau ,g_{1,\infty }\right),$$

$$\theta_{G}\left(B,\tau ,g_{1,\infty }\right)=\alpha_{G}\left(B,g_{1,\infty }\right)\cap \epsilon_{G}\left(B,\tau ,g_{1,\infty }\right)$$

and

$$\theta_{G}\left(B,g_{1,\infty }\right)=\alpha_{G}\left(B,g_{1,\infty }\right)\cap \epsilon_{G}\left(B,g_{1,\infty }\right).$$

**Theorem** **3.** *Assume that G is a Furstenberg family, and that $\left(Y,g_{1,\infty }\right)$ is a T-VDDS. If G is compatible with the system $\left(Y,g_{1,\infty }\right)$, then the following hold:*

(1) *$\alpha_{G}\left(B,g_{1,\infty }\right)$ is a $G_{\delta }$ set for any B⊂Y.*
(2) *$\theta_{G}\left(B,\tau ,g_{1,\infty }\right)$ is a $G_{\delta }$ set for any B⊂Y and any τ>0.*

**Proof.**  From the condition, the set $G(F,g_{1,\infty })$ is a $G_{\delta }$ set for any open set F⊂Y. As

$$\alpha_{G}\left(B,g_{1,\infty }\right)=\overset{\infty }{\underset{k=1}{⋂}}G\left({\left[B\right]}_{\frac{1}{k}},g_{1,\infty }\right),$$

$\alpha_{G}\left(B,g_{1,\infty }\right)$ is a $G_{\delta }$ set. As

$$\epsilon_{G}\left(B,\tau ,g_{1,\infty }\right)=G\left(Y-\bar{{\left[B\right]}_{\tau }},g_{1,\infty }\right)$$

and

$$\theta_{G}\left(B,\tau ,g_{1,\infty }\right)=\alpha_{G}\left(B,g_{1,\infty }\right)\cap \epsilon_{G}\left(B,\tau ,g_{1,\infty }\right),$$

$\theta_{G}\left(B,\tau ,g_{1,\infty }\right)$ is a $G_{\delta }$ set. Thus, the proof is ended. □

Let C,D⊂Y. *C* and *D* are said to be positively disjoint if ρ(C,D) is positive.

**Theorem** **4.** *Assume that A,B⊂Y, A, and B are positively disjoint, G is a Furstenberg family, and $\left(Y,g_{1,\infty }\right)$ is a T-VDDS. If G is compatible with the system $\left(Y,g_{1,\infty }\right)$, then the following holds:*

(1) *$\alpha_{G}\left(B,g_{1,\infty }\right)\subset \epsilon_{G}\left(A,\tau ,g_{1,\infty }\right)$ for some τ>0.*
(2) *$\theta_{G}\left(A,\tau ,g_{1,\infty }\right)⊃\alpha_{G}\left(A,g_{1,\infty }\right)\cap \alpha_{G}\left(B,g_{1,\infty }\right)$ for some τ>0.*

**Proof.**  As *A* and *B* are positively disjointed, there is some τ>0 with $\bar{{\left[A\right]}_{\tau }}\cap \bar{{\left[B\right]}_{\tau }}=\emptyset$. So, one has

$$\alpha_{G}\left(B,g_{1,\infty }\right)=\underset{\varepsilon >0}{⋂}\underset{G\in G}{⋃}\underset{n\in G}{⋂}{\left(g_{1}^{n}\left({\left[B\right]}_{\tau }\right)\right)}^{-1}\subset \underset{G\in G}{⋃}\underset{n\in G}{⋂}{\left(g_{1}^{n}\left({\left[B\right]}_{\tau }\right)\right)}^{-1}\subset \underset{G\in G}{⋃}\underset{n\in G}{⋂}{\left(g_{1}^{n}\left(Y-\bar{{\left[A\right]}_{\tau }}\right)\right)}^{-1}.$$

This implies that

$$\alpha_{G}\left(B,g_{1,\infty }\right)\subset \epsilon_{G}\left(A,\tau ,g_{1,\infty }\right)$$

and $\theta_{G}\left(A,\tau ,g_{1,\infty }\right)⊃\alpha_{G}\left(A,g_{1,\infty }\right)\cap \alpha_{G}\left(B,g_{1,\infty }\right)$. □

**Theorem** **5.** *Assume that A⊂Y is nonempty, G is a Furstenberg family, and $\left(Y,g_{1,\infty }\right)$ bis a T-VDDS. If G is compatible with the system $\left(Y,g_{1,\infty }\right)$, then one can find B⊂Y with B≠∅ and ρ(A,B)>0, satisfying that $\alpha_{G}\left(A,g_{1,\infty }\right)$ and $\alpha_{G}\left(B,g_{1,\infty }\right)$ are dense in Y, if and only if $\theta_{G}\left(A,\varepsilon ,g_{1,\infty }\right)$ is a $G_{\delta }$ dense set in Y for some ε>0.*

**Proof.**  Assume that there is a B⊂Y with B≠∅, ρ(A,B)>0, and $\alpha_{G}\left(A,g_{1,\infty }\right)$ and $\alpha_{G}\left(B,g_{1,\infty }\right)$ are dense in *Y*. From Theorem 3, $\alpha_{G}\left(A,g_{1,\infty }\right)$ and $\alpha_{G}\left(B,g_{1,\infty }\right)$ are $G_{\delta }$ dense sets in *Y*. From Theorem 4, $\theta_{G}\left(A,\varepsilon ,g_{1,\infty }\right)⊃\alpha_{G}\left(A,g_{1,\infty }\right)\cap \alpha_{G}\left(B,g_{1,\infty }\right)$ for some ε>0. So, from Theorem 3, $\theta_{G}\left(A,\varepsilon ,g_{1,\infty }\right)$ is a $G_{\delta }$ dense set in *Y* for the above ε>0. □

Now, we assume that $\theta_{G}\left(A,\varepsilon ,g_{1,\infty }\right)$ is a $G_{\delta }$ dense set in *Y* for some ε>0. As $\alpha_{G}\left(A,g_{1,\infty }\right)⊃\theta_{G}\left(A,\varepsilon ,g_{1,\infty }\right)$, $\alpha_{G}\left(A,g_{1,\infty }\right)$ is a dense set in *Y*. Let $B=Y-{\left[A\right]}_{\varepsilon }$. Then, B≠∅, ρ(A,B)>0 and $\alpha_{G}\left(B,g_{1,\infty }\right)⊃\theta_{G}\left(A,\varepsilon ,g_{1,\infty }\right)$. So, $\alpha_{G}\left(B,g_{1,\infty }\right)$ is a dense set in *Y*.

### 3.3. Chaos with Respect to a Furstenberg Family for a T-VDDS

For a given T-VDDS $\left(Y,g_{1,\infty }\right)$, an G-reciprocating point (resp. G-τ-reciprocating point) of the diagonal $\Delta_{Y}$ for the product system $\left(Y\times Y,g_{1,\infty }\times g_{1,\infty }\right)$ is called an G-scrambled pair (resp. G-τ-scrambled pair) of the system $\left(Y,g_{1,\infty }\right)$. A subset S⊂Y is called an G-scrambled scrambled set for a given T-VDDS $\left(Y,g_{1,\infty }\right)$ if any (u,v)∈S×S(u≠v) is an G-scrambled pair of this system. A subset S⊂Y is called a strongly G-scrambled scrambled set for a given T-VDDS $\left(Y,g_{1,\infty }\right)$ if there is a τ>0, such that any two different points in *S* is an G-τ-scrambled pair of this system. A given T-VDDS $\left(Y,g_{1,\infty }\right)$ is called generically G-chaotic if the set of all G-scrambled pairs of this system is a $G_{\delta }$ set, which is dense in Y×Y. A given T-VDDS $\left(Y,g_{1,\infty }\right)$ is called generically strongly G-chaotic if there is a τ>0 that satisfies that the set of all G-τ-scrambled pairs of this system is a $G_{\delta }$ set, which is dense in Y×Y.

**Theorem** **6.** *Assume that τ>0 and $\left(Y,g_{1,\infty }\right)$ are a T-VDDS. Then, the following hold:*

(1) *A pair (u,v) of points in Y is an $\bar{M}\left(0\right)$-scrambled pair (resp. $\bar{M}\left(0\right)$-τ-scrambled pair) of the system $\left(Y,g_{1,\infty }\right)$ if and only if the pair is a scrambled pair (resp. τ-scrambled pair) in the Li–Yorke sense for the system $\left(Y,g_{1,\infty }\right)$.*
(2) *A set S⊂Y is an $\bar{M}\left(0\right)$-scrambled (resp. strongly $\bar{M}\left(0\right)$-scrambled) set for the system $\left(Y,g_{1,\infty }\right)$, if and only if the set is a scrambled set (resp. strongly scrambled set) in the Li–Yorke sense for the system $\left(Y,g_{1,\infty }\right)$.*
(3) *A pair (u,v) of points in Y is an $\bar{M}\left(1\right)$-scrambled pair (resp. $\bar{M}\left(1\right)$-τ-scrambled pair) of the system $\left(Y,g_{1,\infty }\right)$, if and only if the pair is a scrambled pair (resp. τ-scrambled pair) in the distribution sense for the system $\left(Y,g_{1,\infty }\right)$.*
(4) *A set S⊂Y is an $\bar{M}\left(1\right)$-scrambled (resp. strongly $\bar{M}\left(1\right)$-scrambled) set for the system $\left(Y,g_{1,\infty }\right)$, if and only if the set is a scrambled set (resp. strongly scrambled set) in the distribution sense for the system $\left(Y,g_{1,\infty }\right)$.*

**Proof.**  The proof is immediate and omitted. □

### 3.4. Criteria for a Generically Strongly G-Chaotic T-VDDS

**Theorem** **7.** *Assume that G is a Furstenberg family and $\left(Y,g_{1,\infty }\right)$ is a T-VDDS. If G is compatible with the system $\left(Y\times Y,g_{1,\infty }\times g_{1,\infty }\right)$, then the system $\left(Y,g_{1,\infty }\right)$ is generically strongly G-chaotic, if and only if the following hold:*

(1) *$\alpha_{G}\left(\Delta_{Y},g_{1,\infty }\times g_{1,\infty }\right)$ is dense in Y×Y, and*
(2) *There exists a nonempty B⊂Y×Y that is positively disjoint with $\Delta_{Y}$, satisfying that $\alpha_{G}\left(B,g_{1,\infty }\times g_{1,\infty }\right)$ is dense in Y×Y.*

**Proof.**  The proof is direct and omitted for brevity. □

**Theorem** **8.** *Assume that G is a Furstenberg family, $\left(Y,g_{1,\infty }\right)$ is a T-VDDS, and A,C⊂Y. Then,*

$$\alpha_{G}\left(A\times C,g_{1,\infty }\times g_{1,\infty }\right)⊃\alpha_{\kappa B}\left(A,g_{1,\infty }\right)\times \alpha_{G}\left(C,g_{1,\infty }\right).$$

**Proof.**  For any τ>0, choose $\tau_{1}>0$ with ${\left[A\right]}_{\tau_{1}}\times {\left[C\right]}_{\tau_{1}}\subset {\left[A\times C\right]}_{\tau }$. Let

$$\left(u,v\right)\in \alpha_{\kappa B}\left(A,g_{1,\infty }\right)\times \alpha_{G}\left(C,g_{1,\infty }\right).$$

Then, $u\in \alpha_{\kappa B}\left(A,g_{1,\infty }\right)$ and $v\in \alpha_{G}\left(C,g_{1,\infty }\right)$. This means

$$N_{g_{1,\infty }}\left(u,{\left[A\right]}_{\tau_{1}}\right)\in \kappa B$$

and

$$N_{g_{1,\infty }}\left(v,{\left[C\right]}_{\tau_{1}}\right)\in G.$$

From the hypothesis and the definition, one has

$$N_{g_{1,\infty }\times g_{1,\infty }}\left(\left(u,v\right),{\left[A\times C\right]}_{\tau }\right)⊃N_{g_{1,\infty }\times g_{1,\infty }}\left(\left(u,v\right),{\left[A\right]}_{\tau_{1}}\times {\left[C\right]}_{\tau_{1}}\right)⊃N_{g_{1,\infty }}\left(u,{\left[A\right]}_{\tau_{1}}\right)\cdot N_{g_{1,\infty }}\left(v,{\left[C\right]}_{\tau_{1}}\right)\in G.$$

Consequently, $\left(u,v\right)\in \alpha_{G}\left(A\times C,g_{1,\infty }\times g_{1,\infty }\right)$. □

**Theorem** **9.** *Assume that G is a full Furstenberg family, and that $\left(Y,g_{1,\infty }\right)$ is a T-VDDS, and let G be compatible with the product system $\left(Y\times Y,g_{1,\infty }\times g_{1,\infty }\right)$. If there are a point p∈Y and a A⊂Y with A≠∅ and ρ(p,A)>0, such that $\alpha_{\kappa B}\left(p,g_{1,\infty }\right)$ and $\alpha_{G}\left(A,g_{1,\infty }\right)$ are dense, then the system $\left(Y,g_{1,\infty }\right)$ is generically strongly G-chaotic.*

**Proof.**  From the hypothesis and Theorem 8, one can see that

$$\alpha_{\kappa B}\left(\left\{\left(p,p\right)\right\},g_{1,\infty }\times g_{1,\infty }\right)$$

is dense in Y×Y, and that

$$\alpha_{\kappa B}\left(\left\{p\right\}\times A,g_{1,\infty }\times g_{1,\infty }\right)\;$$

is dense in Y×Y. As G is full,

κB⊂G.

Therefore,

$$\alpha_{G}\left(\left\{\left(p,p\right)\right\},g_{1,\infty }\times g_{1,\infty }\right)\;$$

is dense in Y×Y. Clearly,

$${\left[\Delta_{Y}\right]}_{\tau }⊃{\left[\left\{\left(p,p\right)\right\}\right]}_{\tau }\;$$

for any τ>0. So,

$$\alpha_{G}\left(\left\{\left(p,p\right)\right\},g_{1,\infty }\times g_{1,\infty }\right)\subset \alpha_{G}\left(\left[\Delta_{Y}\right],g_{1,\infty }\times g_{1,\infty }\right).\;$$

Hence,

$$\alpha_{G}\left(\left[\Delta_{Y}\right],g_{1,\infty }\times g_{1,\infty }\right)\;$$

is dense in Y×Y. From Theorem 7, the system $\left(Y,g_{1,\infty }\right)$ is generically strongly G-chaotic. □

**Theorem** **10.** *Assume that $\left(Y,g_{1,\infty }\right)$ is a T-VDDS, p∈Y is a fixed point such that $\overset{\infty }{\underset{j=1}{⋃}}{\left(g_{1}^{j}\left(\left\{p\right\}\right)\right)}^{-1}$ is dense, and B⊂Y is invariant with B≠∅, ρ(p,B)>0, and $\overset{\infty }{\underset{j=1}{⋃}}{\left(g_{1}^{j}\left(A\right)\right)}^{-1}$ is dense. If G is a full Furstenberg family that is compatible with the system $\left(Y\times Y,g_{1,\infty }\times g_{1,\infty }\right)$, then the system $\left(Y,g_{1,\infty }\right)$ is generically strongly G-chaotic. Consequently, the system $\left(Y,g_{1,\infty }\right)$ is generically strongly $\bar{M}\left(1\right)$-chaotic.*

**Proof.**  From the hypothesis, we can see that

$$\alpha_{\kappa B}\left(\left\{p\right\},g_{1,\infty }\right)\;$$

is dense in *Y*, and that

$$\alpha_{\kappa B}\left(A,g_{1,\infty }\right)\;$$

is dense in *Y*. As G is full,

κB⊂G.

Therefore,

$$\alpha_{G}\left(A,g_{1,\infty }\right)\;$$

is dense in *Y*. From Theorem 9, the system $\left(Y,g_{1,\infty }\right)$ is generically strongly G-chaotic. From Theorems 2 and 9, the system $\left(Y,g_{1,\infty }\right)$ is generically strongly $\bar{M}\left(1\right)$-chaotic. □

For any given integer m≥2, we let the set $N_{m}=\left\{1,2,\cdots ,m\right\}$ be endowed with the discrete topology and the space $\Sigma_{m}=\prod_{j=1}^{\infty }N_{m}$ be endowed with the product topology. The compact metric space $\Sigma_{m}$ is said to be the symbolic space generated by *m* different elements. The shift map σ on the space $\Sigma_{m}$ is defined by $\sigma \left(y_{1}y_{2}\cdots \right)=y_{2}y_{3}\cdots$ for any $y_{1}y_{2}\cdots \in \Sigma_{m}$. Obviously, $11\cdots \in \Sigma_{m}$ and $22\cdots \in \Sigma_{m}$ are the fixed points of the shift map $\sigma_{j}$, where $\sigma_{j}=\sigma^{j}\circ \sigma^{j-1}\circ \cdots \circ \sigma^{2}\circ \sigma =\sigma^{\frac{j(j+1)}{2}}$ for every integer j≥1. Then, we obtain a T-VDDS $\left(\Sigma_{m},\sigma_{1,\infty }\right)$, given by sequences ${\left(\sigma_{j}\right)}_{j=1}^{\infty }$ of continuous maps $\sigma_{j}$. As an application, we have the following example.

**Example** **1.** *If G is a full Furstenberg family that is compatible with the product system $\left(\Sigma_{M}\times \Sigma_{M},\sigma_{1,\infty }\times \sigma_{1,\infty }\right)$, then the system $\left(\Sigma_{M},\sigma_{1,\infty }\right)$ is generically strongly G-chaotic. Consequently, the system $\left(\Sigma_{M},\sigma_{1,\infty }\right)$ is generically strongly $\bar{M}\left(1\right)$-chaotic.*

**Proof.**  Clearly, $11\cdots \in \Sigma_{m}$ and $22\cdots \in \Sigma_{m}$ are the fixed points of the system $\sigma_{1,\infty }$. Also, it is easy to show that the conditions of Theorem 10 are satisfied for these two fixed points. From Theorem 10, the result of Example 1 holds. □

**Remark** **1.** *For any countably infinite set of functions $\left\{g_{1},g_{2},\cdots \right\}$, in order for results to be independent of the numbering, we need a condition on the family G, namely that if B∈G, then H(B)∈G for any permutation of the set of positive integers.*

## 4. Conclusions

In recent years, numerous researchers have delved into T-VDDSs and made significant progress. This study utilizes the Furstenberg family theory to establish G-scrambled pairs for a T-VDDS and explores certain characteristics of the collection of G-scrambled pairs. Additionally, we introduce the concept of generically G-chaotic T-VDDSs and generically strongly G-chaotic T-VDDSs, establish a condition sufficient for T-VDDSs to be generically strongly G-chaotic, and present an example of a generically strongly $\bar{M}\left(1\right)$-chaotic T-VDDS. Our future research will focus on the practical applications of these systems.

## Data Availability

The data presented in this study are available on request from the corresponding author.
